# COVID-19 atypical Parsonage-Turner syndrome: a case report

**DOI:** 10.1186/s12883-022-02622-4

**Published:** 2022-03-16

**Authors:** Maria Beatrice Zazzara, Anna Modoni, Alessandra Bizzarro, Alessandra Lauria, Francesca Ciciarello, Cristina Pais, Vincenzo Galluzzo, Francesco Landi, Matteo Tostato, Gemelli Against COVID-19 Post-Acute Care Team, Francesco Landi, Elisa Gremese, Roberto Bernabei, Massimo Fantoni, Antonio Gasbarrini, Carlo Romano Settanni, Serena Porcari, Francesca Benvenuto, Giulia Bramato, Vincenzo Brandi, Angelo Carfì, Francesca Ciciarello, Maria Rita Lo Monaco, Anna Maria Martone, Emanuele Marzetti, Carmen Napolitano, Vincenzo Galluzzo, Francesco Pagano, Cristina Pais, Sara Rocchi, Elisabetta Rota, Andrea Salerno, Matteo Tosato, Marcello Tritto, Riccardo Calvani, Maria Beatrice Zazzara, Lucio Catalano, Anna Picca, Giulia Savera, Roberto Cauda, Rita Murri, Antonella Cingolani, Giulio Ventura, Eleonora Taddei, Davide Moschese, Arturo Ciccullo, Massimo Fantoni, Leonardo Stella, Giovanni Addolorato, Francesco Franceschi, Gertrude Mingrone, Maria Assunta Zocco, Maurizio Sanguinetti, Paola Cattani, Simona Marchetti, Brunella Posteraro, Michela Sali, Alessandra Bizzarro, Alessandra Lauria, Stanislao Rizzo, Maria Cristina Savastano, Gloria Gambini, Maria Grazia Cozzupoli, Carola Culiersi, Giulio Cesare Passali, Gaetano Paludetti, Jacopo Galli, Fabrizio Crudo, Giovanni Di Cintio, Ylenia Longobardi, Laura Tricarico, Mariaconsiglia Santantonio, Tiziana Di Cesare, Mariateresa Guarino, Marco Corbò, Stefano Settimi, Dario Mele, Francesca Brigato, Danilo Buonsenso, Piero Valentini, Dario Sinatti, Gabriella De Rose, Luca Richeldi, Francesco Lombardi, Angelo Calabrese, Francesco Varone, Paolo Maria Leone, Matteo Siciliano, Giuseppe Maria Corbo, Giuliano Montemurro, Mariarosaria Calvello, Enrica Intini, Jacopo Simonetti, Giuliana Pasciuto, Veronica Adiletta, Carmelo Sofia, Maria Angela Licata, Gabriele Sani, Delfina Janiri, Alessio Simonetti, Marco Modica, Silvia Montanari, Antonello Catinari, Beatrice Terenzi, Luigi Natale, Anna Rita Larici, Riccardo Marano, Tommaso Pirronti, Amato Infante, Annamaria Paglionico, Luca Petricca, Barbara Tolusso, Stefano Alivernini, Clara Di Mario, Angelo Santoliquido, Luca Santoro, Antonio Nesci, Angela Di Giorgio

**Affiliations:** 1 Department of Gerontology, Neuroscience and Orthopedics, Fondazione Universitaria Policlinico Gemelli, IRCCS, Rome, Italy; 2 Unità Operativa Complessa Di Neurologia, Fondazione Policlinico Universitario A. Gemelli IRCCS, Rome, Italy

**Keywords:** Sars-CoV-2 infection, Atypical brachial plexus neuritis, COVID-19 neuromuscular sequelae, COVID-19 neurological manifestations

## Abstract

**Background:**

Neurological manifestations of Sars-CoV-2 infection have been described since March 2020 and include both central and peripheral nervous system manifestations. Neurological symptoms, such as headache or persistent loss of smell and taste, have also been documented in COVID-19 long-haulers. Moreover, long lasting fatigue, mild cognitive impairment and sleep disorders appear to be frequent long term neurological manifestations after hospitalization due to COVID-19. Less is known in relation to peripheral nerve injury related to Sars-CoV-2 infection.

**Case presentation:**

We report the case of a 47-year-old female presenting with a unilateral chest pain radiating to the left arm lasting for more than two months after recovery from Sars-CoV-2 infection. After referral to our post-acute outpatient service for COVID-19 long haulers, she was diagnosed with a unilateral, atypical, pure sensory brachial plexus neuritis potentially related to COVID-19, which occurred during the acute phase of a mild Sars-CoV-2 infection and persisted for months after resolution of the infection.

**Conclusions:**

We presented a case of atypical Parsonage-Turner syndrome potentially triggered by Sars-CoV-2 infection, with symptoms and repercussion lasting after viral clearance. A direct involvement of the virus remains uncertain, and the physiopathology is unclear. The treatment of COVID-19 and its long-term consequences represents a relatively new challenge for clinicians and health care providers. A multidisciplinary approach to following-up COVID-19 survivors is strongly advised.

## Background

The coronavirus disease 2019 (COVID-19) pandemic, caused by the severe acute respiratory syndrome coronavirus 2 (Sars-CoV-2), has crucially affected the life of everyone worldwide and significantly changed the course of history. Whereas typical symptoms of COVID-19 involve mainly the respiratory system, neurological manifestations have been described since March 2020 [1]. In a retrospective study of 214 hospitalised patients with COVID-19, authors identified both central nervous system (CNS) and peripheral nervous system (PNS) manifestations [2]. CNS manifestations included dizziness, headache, impaired consciousness and seizures, vision impairment, while PNS included anosmia or dysgeusia, later identified as two cardinal symptoms of this diseases, but also neuropathic pain [2]. Furthermore, over the past two year, more complicated neurological manifestations, such as Guillain-Barré syndrome, have been reported in patients affected by COVID-19 [3]. Neurological manifestations were more common in severe cases or in those with prolonged hospitalization [2].

Neurological symptoms, such as headache or persistent loss of smell and taste, have also been documented in COVID-19 long-haulers [4]. Long lasting fatigue, mild cognitive impairment and sleep disorders also seem to be frequent long term neurological manifestations after hospitalization due to COVID-19 and seem to be significantly related to the severity of respiratory symptoms, similarly to the acute phase [5]. However, little is known in relation to peripheral nerve injury in patients recovered from COVID-19. Hereby we report a case of atypical brachial plexitis in a patient admitted to our post-acute outpatient service “The Gemelli Against COVID-19 Post-Acute Care (GAC19-PAC)” for COVID-19 survivors at Fondazione Policlinico Universitario Agostino Gemelli IRCCS in Rome, Italy.

### Case representation

Our patient is a 47-year-old female nurse affected by chronic gastritis and gastroesophageal reflux disease for which she cyclically takes proton pump inhibitors.

On November the 2^nd^ 2020, after having returned home from a hospital shift, she started feeling extremely tired and developed flu-like symptoms including cough, myalgia and loss of appetite. Two days later, a real time-polymerase chain reaction (RT-PCR) of nasopharyngeal swab test confirmed that she had contracted Sars-CoV-2 infection. While in self-isolation, she started developing anosmia and dysgeusia and difficulties in sleeping. It was only when she manifested shortness of breath and chest pain that she was admitted to the emergency department of a large hospital in Rome. In addition to dyspnoea, she refers to have experienced an acute and intense burning chest pain radiating to the proximal left arm, the shoulder and the upper region of the homolateral hemithorax, innerved by T1 root. A chest computed tomography scan (CT-scan) showed that she had developed a mild bilateral pneumonia Sars-CoV-2 related, without respiratory insufficiency, but could not explain the peculiar chest pain. Other causes of chest pain were ruled out and she was discharged home on oral steroids with great benefit not only for the respiratory symptoms but also for the pain. On November 21^st^, 2020, a new RT-PCR of nasopharyngeal swab gave a negative result.

About two months later, she was admitted to our post-acute outpatient service as a COVID-19 long hauler, referring the persistence of the unilateral chest pain radiating to the left arm and altered sense of smell and taste along with persistent fatigue and myalgia.

During her three separate days admission to our post-acute Day Hospital service, the patient performed different tests in the three different visits she attended. Overall, she undertook a full blood test panel including autoimmune markers without abnormalities, an electrocardiogram revealing a sinus rhythm, a pneumological examination with full pulmonary tests displaying normal lung function, a psychological evaluation, and a neurological examination. The autoimmune markers panel included Anti-nuclear autoantibodies (ANA), Anti-SSA/Ro, Anti-La/SSB, Anti-Jo1, Anti-RNP; Anti-topoisomerase I (Anti-SCL 70); Central-antineutrophil cytoplasmatic antibody (c-ANCA); Perinuclear-antineutrophil cytoplasmatic antibody (p-ANCA); Rheumatoid factor; Anti-cyclin citrullinated peptide (Anti-CCP); Lupus Anticoagulant; Anti-cardiolipin; Anti-beta2 glycoprotein; Anti-endomysium and Anti-tissue transglutaminase autoantibodies.

**Fig. 1:**
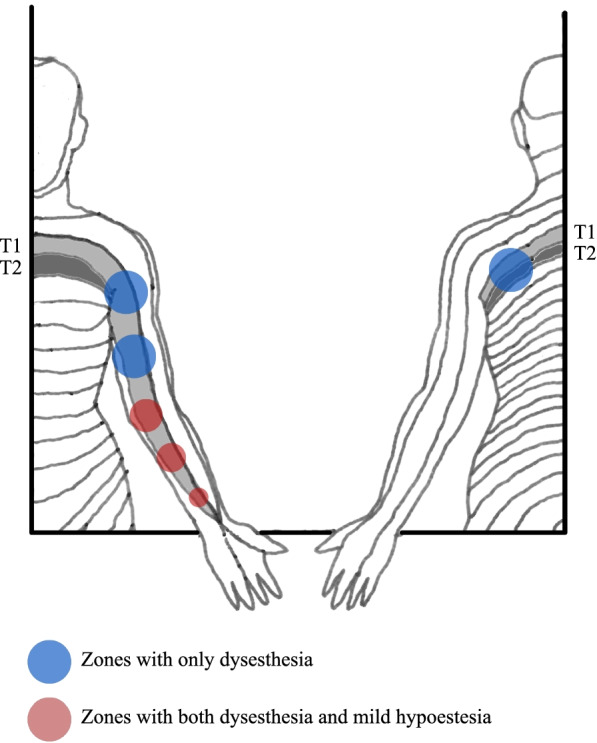
Presentation of patient’s neuropathic symptoms as captured by the neurological physical examination. In light grey: T1 dermatome. In dark grey: T2 dermatome. In blue: areas characterized by dysesthesia. In red: areas characterized by both dysesthesia and mild hypoesthesia. Image was hand produced and graphically elaborated by Lorenzo Di Stefano on behalf of all authors

The neurological physical examination revealed the persistence of painful dysesthesia involving the left scapula, radiating to the left arm and forearm, especially on the ventral side. Muscle strength was normal. A mild hypoesthesia was present in the ventral area of the left arm, with T1 dermatome distribution. The rest of neurological examination did not report any abnormalities. Presentation of patient’s neuropathic symptoms as captured by the neurological physical examination is shown in Fig. 1.

Thus, she performed a neurophysiological evaluation and a magnetic resonance imaging (MRI) of the brachial plexus. MRI was carried out without contrast. Sensory nerve conduction performed on both arms showed a reduced sensory action potential amplitude of the left medial and lateral antebrachial cutaneous nerves, compared to the contralateral, asymptomatic side, with an amplitude difference of more than 50%. Motor nerve conduction studies were unremarkable. Needle electromyography (EMG) of the left upper limb, including deltoid, biceps brachii, extensor digitorum communis and first dorsal interosseus muscles was normal.

**Fig. 2:**
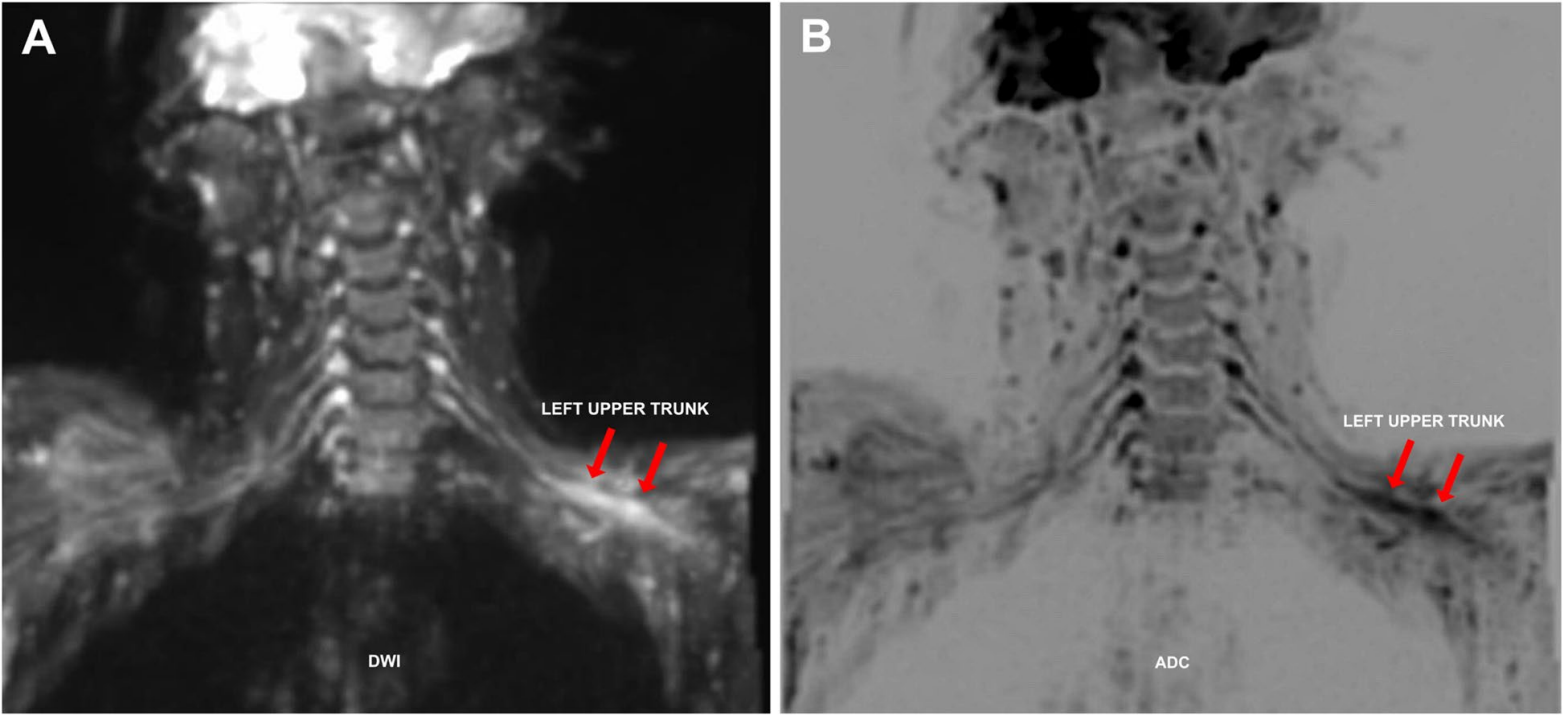
**(A-B):** DWI coronal scan showing segmental DWI-restriction of the left upper trunk (proximal–distal extension:6 cm) (**A**), confirmed at ADC-map (**B**)

**Fig. 3:**
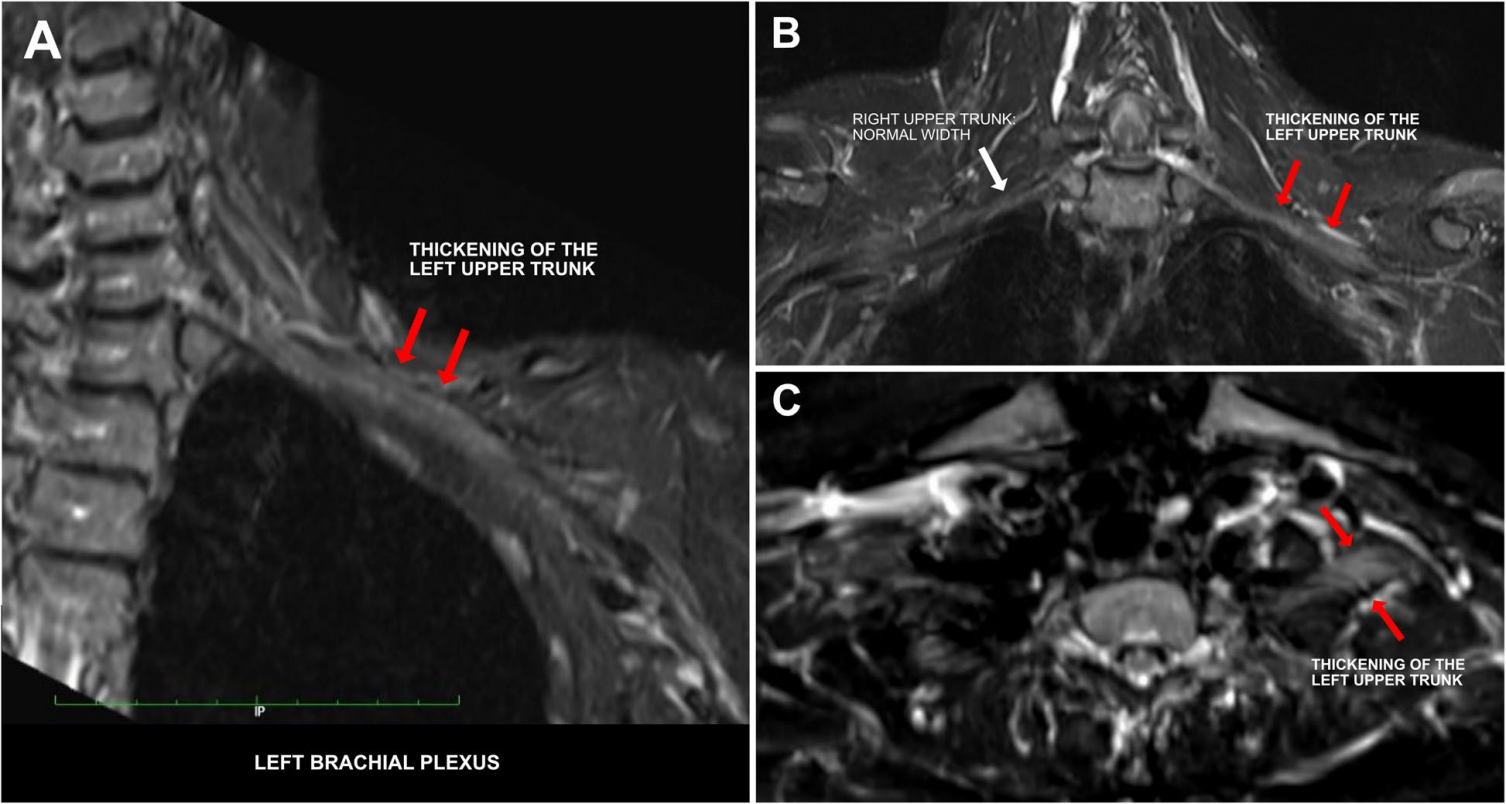
**(A-B-C)**: T2-weighted short-tau inversion recovery (STIR) MRI sequences showing corresponding hyperintensity and thickening of the left upper trunk

**Fig. 4:**
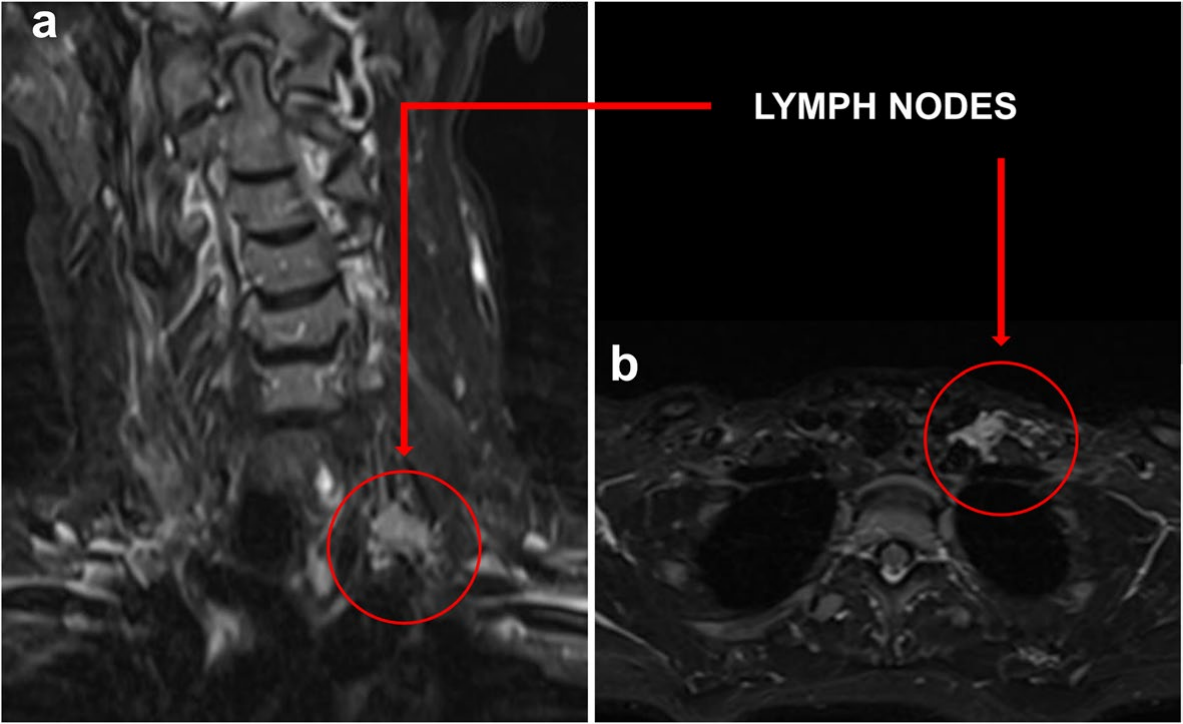
**(a-b)**: T2 STIR coronal scan showing some confluent lymph nodes with irregular borders in left supraclavicular fossa

The MRI demonstrated segmental diffusion-weighted imaging (DWI) restriction and corresponding apparent diffusion coefficient (ADC) low signal of the left upper trunk (Fig. 2), and T2 hyperintensity and thickening of the left upper trunk (Fig. 3). Some confluent lymph nodes with irregular borders were detected in left supraclavicular fossa (Fig. 4).

A diagnosis of atypical, pure sensory, brachial plexus neuritis was made and she was prescribed to continue oral steroids—at the dosage 12,5 mg of prednisone per day for other two months- and start duloxetine and gabapentin with a consequent significant improvement of the pain, undeterred by mild symptoms under stress.

## Discussion and conclusions

Despite still representing a threat to our health and a challenge to our health care systems, much has improved in the treatment of this novel type of disease since the beginning of the pandemic, with an increased number of COVID-19 survivors. Recovery from Sars-CoV-2 infection is extremely variable among individuals and can significantly affect the health and quality of life of different people. Long-term sequalae related to neurological manifestation and neuromuscular disorders have become more apparent [3].

We presented a 47-year-old female with an atypical brachial plexitis related to a moderate form of Sars-CoV-2 infection, that manifested a peculiar involvement of sensory fibres of both the upper trunk/lateral cord and the lower trunk/medial cord of the left brachial plexus, extending also to T1 dermatome region, whilst sparing motor fibres conversely to other reported cases [6, 7]. The majority of the described peripherical nerve abnormalities have been correlated to the severity of the acute critical illness or related to prolonged patient prone positioning during hospitalization [3, 8]. Recently, a pure sensory involvement of the left brachial plexus was described by Cacciavillani et al. in a 52-years-old patient who developed neuralgic symptoms a week after hospitalization for COVID-19 pneumoniae, complicated with respiratory insufficiency [9]. Our patient, however, was not exposed to any of the potential triggers as she did not experience a prolonged hospitalization nor prone positioning nor a severe illness. Nevertheless, because a cerebrospinal fluid examination was not performed nor a nerve or a lymph node biopsy, mechanisms underlying the pathophysiology of the presented atypical brachial plexitis are uncertain and whether there could be a direct or indirect viral involvement is questionable.

Parsonage–Turner syndrome (brachial plexus neuritis) or neuralgic amyotrophy is rare peripheral neuropathy characterized by sudden onset of extreme pain, paresis and sensory loss in a brachial plexus distribution and a slow recovery requiring months to years that can also involve other PNS regions [10]. Genetic susceptibility, autoimmune disorders, infectious disease, and recent vaccination have been correlated to the aetiology and pathophysiology of neuralgic amyotrophy, although it still remains unclear [11]. A case of Parsonage-Turner syndrome has been reported in a asymptomatic 17-year-old female with positive serum IgG antibodies for Sars-CoV-2 [11] and a 50 years-old healthy male one week after receiving the first shot of the Comirnaty vaccine [12]. Other viral infections – including herpes simplex virus and varicella zoster, Epstein-Barr virus, cytomegalovirus, HIV, Hepatitis B [11] – have been documented to precede neuralgic amyotrophy. Similarly, Sars-CoV-2 could directly involve the brachial plexus or induce and aberrant autoimmune/inflammatory response, leading to neuralgic symptoms.

Other coronaviruses have demonstrated a significant neurotropism [13] and Sars-CoV-2 could trigger a similar response, potentially involving the brachial plexus already during the acute phase, triggering the onset of the plexitis. Mechanisms explaining the neurological involvement related to COVID-19 are not fully understood. The virus could reach the nervous system via a compromised blood–brain barrier, or migrate via neuronal pathways, infecting sensory or motor nerve endings, leading to subsequent neurological damage and neuroinflammation [13, 14]. It has been suggested that the angiotensin-converting enzyme 2 (ACE2), identified as the functional receptor for Sars-CoV2, which is also expressed in nervous system [15], may be implicated in the COVID-19–related neuropathy [2, 14], although further research is needed to better elucidate the pathophysiology of the neurological involvement.

Our understating of this new type of coronavirus has greatly improved since the beginning of the pandemic, and so has our approach to the acute phase. However, the management of COVID-19 and its potential long-term sequalae after viral clearance still represent a challenge for clinicians and health care providers. COVID-19 and its neurological implications remain an ongoing learning topic for stumbling researchers and clinicians and a comprehensive multidisciplinary follow-up, as in the case of our post-acute day hospital, is crucial for the optimal care of these individuals to help capturing important patients’ needs, nuances and expectation after recovery form Sars-CoV-2 infection.

## Data Availability

All the data and material are available upon request to the study PIs. The corresponding author can be directly contacted to apply for permissions to obtain access to the raw data.

## Abbreviations

COVID-19: Coronavirus disease 2019
Sars-CoV-2: Severe acute respiratory syndrome coronavirus 2
CNS: Central nervous system
PNS: Peripheral nervous system
GAC19-PAC: The Gemelli Against COVID-19 Post-Acute Care
RT-PCR: Real Time-Polymerase Chain Reaction
CT-scan: Computed tomography scan
MRI: Magnetic resonance imaging
EMG: Electromyography
DWI: Diffusion-weighted imaging
HIV: Human immunodeficiency virus
ACE2: Angiotensin-converting enzyme 2
T2-STIR: T2-weighted short-tau inversion recovery
ADC: Apparent diffusion coefficient
ANA: Anti-nuclear autoantibodies
Anti-SCL 70: Anti-topoisomerase I
c-ANCA: Central-antineutrophil cytoplasmatic antibody
p-ANCA: Perinuclear-antineutrophil cytoplasmatic antibody
Anti-CCP: Anti-cyclin citrullinated peptide
